# Evaluation of Programmed Death-Ligand 1 (PD-L1) Expression and Its Correlation With Clinicopathological Parameters in Oral Squamous Cell Carcinoma: A Tertiary Care Center Study

**DOI:** 10.7759/cureus.85186

**Published:** 2025-06-01

**Authors:** Pallavi Srivastava, Nidhi Anand, Deeksha Agarwal, Roopali Upadhyay, Saumya Shukla, Vikas Sharma

**Affiliations:** 1 Pathology, Sanjay Gandhi Post Graduate Institute of Medical Sciences, Lucknow, IND; 2 Pathology, Dr. Ram Manohar Lohia Institute of Medical Sciences, Lucknow, IND; 3 Surgical Oncology, Dr. Ram Manohar Lohia Institute of Medical Sciences, Lucknow, IND

**Keywords:** combined positive score, immunohistochemistry(ihc), oscc, pd-l1 expression, tumor proportion score (tps)

## Abstract

Introduction

This study endeavors to evaluate the frequency of programmed death-ligand 1 (PD-L1) expression in oral squamous cell carcinoma (OSCC) specimens and to correlate these findings with clinicopathological features of established prognostic significance.

Methodology

The study included paired pre-surgical biopsy and resection specimens from 35 patients with OSCC. Immunohistochemistry was performed for PD-L1 (clone SP263) with 51.4% tumor proportion score (TPS) and 77.1% combined proportion score (CPS), which was assessed at a cut-off of ≥1%. The association of the mean expression of the biomarker with clinicopathological parameters has been evaluated.

Results

TPS of PD-L1 expression at the cut-offs of ≥1%, ≥10%, ≥25%, and ≥50%, and the positivity rates were 20% (7/35), 8.6% (3/35), 8.6% (3/35), and 14.3% (5/35), respectively. The CPS at the same cut-offs were 22.9%, 31.4%, 17.1%, 11.4%, and 17.1%, respectively. TPS was significantly correlated with stage (*P* = 0.01), while CPS was associated with perineural invasion (*P* = 0.04) and showed increased expression in the male population (*P* < 0.05).

Conclusions

This study highlights the significance of a larger/resection specimen over a small biopsy for PD-L1 evaluation. Increased PD-L1 expression observed in higher-stage disease, perineural invasion (PNI), and male patients can be validated in a larger cohort and potentially guide the use of anti-PD-L1 therapy in OSCC.

## Introduction

Oral squamous cell carcinoma (OSCC) is the most common malignant oral cancer. In 2022, 389,485 new cases and 188,230 deaths were reported from SCC of the lip and oral cavity [1]. Surgery is the main treatment option for the majority of cases of OSCC, and adjuvant treatment, including radiotherapy and chemotherapy, is limited to cases with distant or locoregional metastasis [2]. In this regard, treatment with immunotherapy has shown promising results against OSCC. However, patient selection is crucial for the determination of the effectiveness of this therapy [3].

After successful clinical trials on anti-programmed death receptor-1 (PD-1) antibodies (pembrolizumab and nivolumab), the U.S. Food and Drug Administration (FDA) has approved immunotherapy as an adjuvant treatment for metastatic SCC of the head and neck region [4-5].

Programmed death-ligand 1 (PD-L1) is known as an inhibitory immune checkpoint in the immune response. It is expressed on tumor cells (TCs) and cells of the tumor microenvironment, including inflammatory cells (activated B- and T-cells). The interaction of PD-L1 with its receptor PD-1 causes the reduced proliferation of PD-1-positive cells. This interaction contributes to immune evasion by modulating T-cell activity, promoting the apoptosis of antigen-specific T-cells, and preventing the apoptosis of regulatory T-cells. Furthermore, the tumor can trigger the expression of PD-L1 on other cells, resulting in decreased T-cell activity in the tumor microenvironment. The PD-L1/PD-1 axis is a key factor in T-cell exhaustion, and checkpoint inhibitors can block this axis and boost the immune system’s response against cancer [6].

PD-L1 is more popular for predicting immunotherapy responses because it can be evaluated using immunohistochemistry (IHC). While PD-1 expression is limited to inflammatory cells, PD-L1 can be detected on both tumor and inflammatory cells [7]. Various studies have used different scoring methods for PD-L1 IHC evaluation, including tumor proportion score (TPS), immune proportion score (IPS), and combined proportion score (CPS). Keynote studies have used TPS for PD-L1 evaluation at different cut-offs [5,8].

In 2020, pembrolizumab, an antibody against PD-1, was approved for use as monotherapy or in combination therapy for patients with a CPS >1% [9]. According to the keynote-040 study that compared TPS and CPS scores, the CPS score in head and neck squamous cell carcinoma (HNSCC) has better therapeutic and clinical implications. The prognostic role of PD-L1 in OSCC has been studied, but the results are conflicting; therefore, the prognostic significance of PD-L1 expression in OSCC remains to be explored [10]. In this study, we aim to evaluate the immunohistochemical expression of PD-L1 using TPS and CPS scores in paired samples of small biopsy and the consecutive resection specimens of OSCC, and correlate the PD-L1 expression with the clinicopathological variables.

## Materials and methods

The study was conducted as a short-term project in the Department of Pathology at a tertiary care hospital from September 2023 to November 2023. The samples received during this period were included prospectively. Additionally, cases from December 2022 to August 2023 were included in the study's retrospective part. A total of 78 cases of OSCC with resection specimens were received during this period. However, 43 cases with resection specimens were excluded from the study per the inclusion and exclusion criteria, and finally, a total of 35 cases were included in the study. The Institutional Research and Institutional Ethical Committee (IEC 113/23) clearance was obtained from Dr. Ram Manohar Lohia Institute of Medical Sciences, Lucknow, India. Informed consent was obtained from all the prospective cases, and a waiver of consent was obtained from all retrospective cases.

The study includes both fresh and archival tissue samples. Fresh tumor tissue samples were obtained from patients undergoing surgery for oral malignancy, and giving consent for the same. Retrospective samples were retrieved from the archives of the Department of Pathology at RMLIMS. Fresh tissues were fixed in formalin and processed routinely into formalin-fixed paraffin-embedded (FFPE) tissue blocks.

Clinical assessment

Adequate clinical details have been documented, including age, sex, personal history, tobacco chewing history, presenting clinical features, duration and course, operative details, and history. Laterality, site, size, appearance, staging, and any site of distant metastasis have been noted. Overall survival (OS) was calculated for all the cases. OS is the time window between diagnosis and death or the last follow-up.

Inclusion and exclusion criteria

The study included all patients who provided written informed consent and were diagnosed with OSCC through histopathology in small biopsy or resection specimens containing viable tumors.

Patients who did not consent to participate, had a history of chemo or radiotherapy before resection, cases which did not have surgery as the first line of treatment, or had biopsies with predominantly necrotic, inadequate, or autolyzed tissue were excluded from the study.

Histopathological evaluation

Hematoxylin and eosin (H&E)-stained sections of each case have been reviewed/examined to confirm and ascertain the histopathological diagnosis and staging as per the 8th edition of the American Joint Committee on Cancer (AJCC) protocols for reporting oral squamous cell cancer, including major prognostic parameters [11].

Immunohistochemistry for PD-L1, assessment, and scoring

Immunohistochemistry (IHC) for PD-L1 was performed on a fully automated Ventana Bench Mark XT (Ventana Medical System Inc., Tucson, AZ) using a ready-to-use rabbit monoclonal anti-PD-L1 antibody, clone SP263 (Cat no. 7494190001) and with the Opti view 3,3-diaminobenzidine (DAB) secondary detection kit (Ventana Medical Systems Inc.). Each batch was run with a positive control (human placenta) and a negative control (obtained by omitting the primary antibody).

Two authors (NA and PS) analyzed all the PD-L1-stained sections for cytoplasmic/membranous staining for PD-L1 in TCs and immune cells (ICs). They assessed the percentage and staining intensity. Staining intensity was scored as +1, +2, or +3. More than 1% staining in the TCs or ICs was positive. All the stained PD-L1 sections were analyzed for TPS, immune proportion score (IPS), and CPS [12].

**Figure 1:**
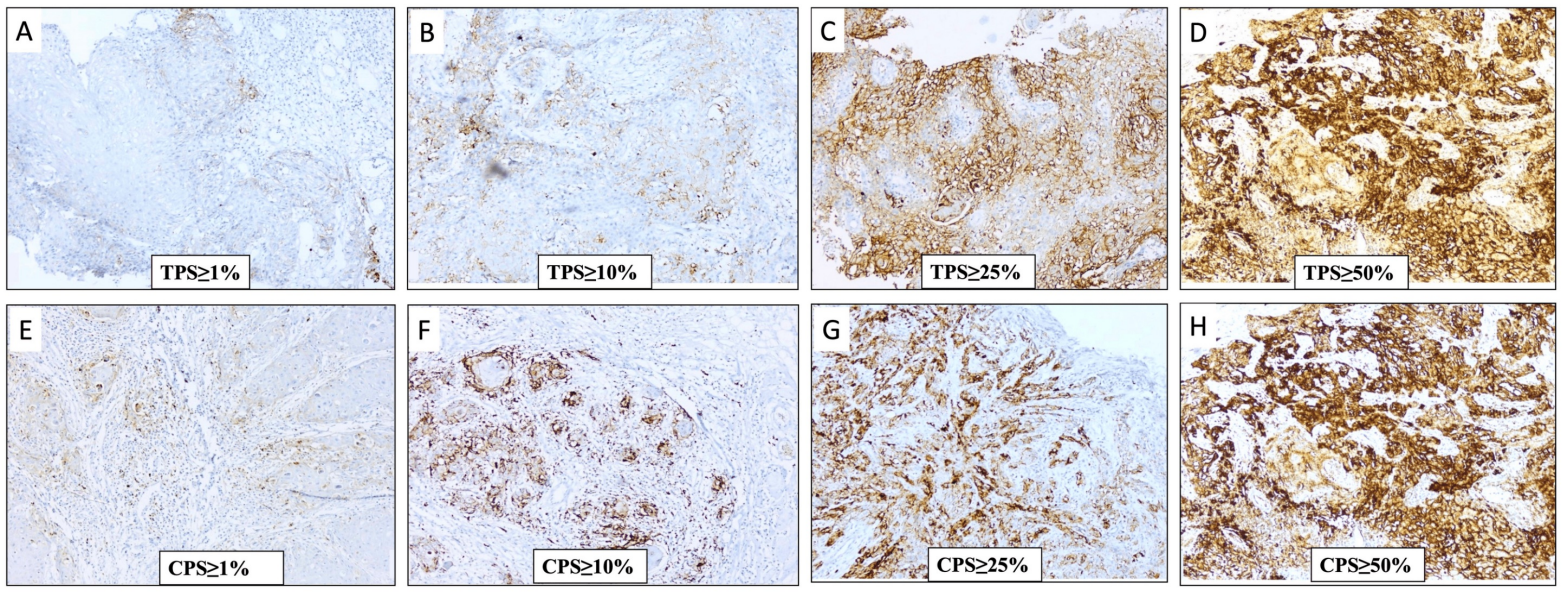
Immunohistochemical expression of PD-L1 in OSCC. (A-D) PD-L1 expression in tumor cells (TPS) at various cut-offs (E-H) PD-L1 expression in tumor and immune cells (CPS) at various cut-offs. (A-H) DAB x200. PD-L1, programmed death-ligand 1; CPS, combined proportion score; TPS, tumor proportion score; OSCC, oral squamous cell carcinoma; DAB, 3,3-diaminobenzidine

TPS was defined as the percentage of viable TCs showing partial or complete membrane staining (≥1+) relative to all viable TCs present in the sample (Figures 1A-1D). IPS was measured as the percentage of PD-L1 expression on immune cells (IC), while CPS combined PD-L1 expression on both TCs and ICs (Figures 1E-1H) [12].

PD-L1 expression in terms of TPS, IPS, and CPS was assessed using cut-off values of ≥1%, ≥10%, ≥25%, and ≥50% (Table 1).

**Table 1:** Comparative analysis of TPS, IPS, and CPS in paired samples. TPS, tumor proportion score; IPS, immune proportion score; CPS, combined proportion score; PD-L1, programmed death-ligand 1

PD-L1 expression	Total paired specimens (*N* = 35)	
	Pre-surgical biopsy, *n* (%)	Surgical resection, *n* (%)
TPS		
Negative	31 (88.6)	17 (48.6)
≥1%	0 (0.0)	7 (20.0)
≥10%	3 (8.6)	3 (8.6)
≥25%	1 (2.9)	3 (8.6)
≥50%	0 (0.0)	5 (14.3)
IPS		
Negative	26 (74.3)	9 (25.7)
≥1%	5 (14.3)	13 (37.1)
≥10%	4 (11.4)	7 (20.0)
≥25%	0 (0.0)	4 (11.4)
≥50%	0 (0.0)	2 (5.7)
CPS		
Negative	26 (74.3)	8 (22.9)
≥1%	3 (8.6)	11 (31.4)
≥10%	5 (14.3)	6 (17.1)
≥25%	1 (2.9)	4 (11.4)
≥50%	0 (0.0)	6 (17.1)

To ensure uniformity in assessment, PD-L1 immunohistochemical expression was initially evaluated in a set of 10 cases by both observers (NA and PS) in consensus. Both screened the rest of the cases independently, blinded to the PD-L1 results of others. This inter-pathologist correlation was checked by applying kappa statistics, and the interobserver variation was 0.93 (excellent agreement), with a significance level of <0.05.

Statistical analysis

The statistical analysis used IBM SPSS, version 21.0 (IBM Corp., Armonk, NY). The frequency of expression of PD-L1 in OSCC was assessed. Contingency tables and chi-square tests were used to correlate PD-L1 IHC results with tumor type, age, gender, pT stage, pN stage, necrosis, depth of invasion, worst pattern of invasion (WPOI), LVI, and PNI, and Pearson correlation was used to find a correlation between paired pre-surgical biopsy specimens and the subsequent resection specimens. A value of *P* < 0.05 was considered statistically significant.

## Results

Clinical characteristics

Participants ranged in age from 25 to 73 years, with an average age of 48.5 years. The male-to-female ratio stood at 3.28:1. Regarding the sites of involvement in the oral cavity, the most frequently affected area was the buccal mucosa, with or without gingivobuccal sulcus, accounting for 48.6% (17/35). This was followed by the tongue at 25.7% (9/35) and the hard palate and alveolus at 8.6% and 5.7%, respectively. T1/T2 stage cases represented 54.2% of the total, while T3/T4 cases made up 45.7% of the cases. N0/N1 cases represented 85.7% of the total, while N2/N3 cases were 14.2 %.

Histopathological characteristics

All 35 cases were histologically verified and diagnosed as squamous cell carcinoma of the oral cavity. Among these, 37.1% (13/35) were classified as well-differentiated, while 62.9% (22/35) were considered moderately differentiated. LVI was observed in 31.4% (11/35) of the cases, and PNI was evident in 20% (7/35). Tumor budding was seen in 82.9% (29/35) of the cases, with WPOI-4 assigned in 48.6% (17/35). The depth of invasion (DOI) analysis showed that >15 mm DOI was noted in 14.2% (5/35), 11-15 mm in 28.6% (10/35), followed by 5-10 mm in 31.4% (11/35), and <5 mm in 25.7% (9/35).

PD-L1 expression in small biopsies

In small biopsies, positive PD-L1 expression was seen in tumor cells in 11.4% (4/35) cases and immune cells in 25.7% (9/35) cases, and CPS was considered positive in 25.7% (9/35) cases.

PD-L1 expression in resection specimens

PD-L1 expression was positive in 51.4% (18/35) of tumor cells and 74.2% (26/35) of cases in immune cells. In 77.1% (27/35) of cases, CPS was given as positive.

**Figure 2:**
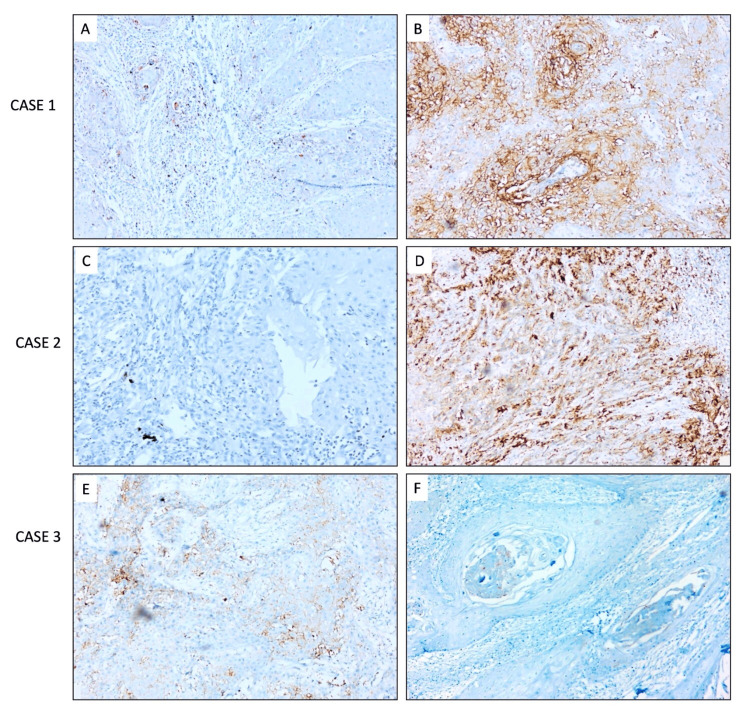
PD-L1 expression in pre-surgical biopsy and surgical resection specimens. (A, B) Case 1 shows positive expressions in both the pre-surgical biopsy (10%) and resection specimens (60%). (C, D) Case 2 shows negative expressions in pre-surgical biopsy (0%) and positive expression in resection specimens (70%). (E, F) Case 3 shows positive expressions in both the pre-surgical biopsy (8%-10%) and negative resection specimens (0%). (A-F) DAB x200. DAB, 3,3-diaminobenzidine; PD-L1, programmed death-ligand 1

A comparative analysis of PD-L1 expression in pre-surgical biopsy and surgical resection specimens showed a poor correlation, as shown in Figures 2A-2F.

Correlation of PD-L1 expression with clinicopathological characteristics

The relationship between TPS and several clinicopathologic parameters was analyzed, revealing a significant association between PD-L1 expression and tumor staging. Higher stages showed increased PD-L1 expression in tumor cells. However, no other parameters were significantly associated (Table 2).

**Table 2:** Correlation between clinicopathological parameters and PD-L1 TPS in OSCC. The chi-square test/Fisher’s exact test was applied as appropriate. *P-value < 0.05 is considered significant. TPS, tumor proportion score; WD, well differentiated; MD, moderately differentiated; PD, poorly differentiated; TILS, tumor infiltrating lymphocytes; T, tumor; LVI, lymphovascular invasion; PNI, perineural invasion; WPOI, worst pattern of invasion; OSCC, oral squamous cell carcinoma; PD-L1, programmed death-ligand 1

Features		Total, *n* (%)	TPS positive (N = 18), *n* (%)	TPS negative (*N *= 17), *n* (%)	*P*-value
Age intervals	≤40 years	9 (25.7)	5 (55.5)	4 (44.4)	0.77
	>40 years	26 (74.3)	13 (50%)	13 (50%)	
Gender	Male	22 (68.1)	14 (63.6)	8 (36.3)	0.06
	Female	13 (31.9)	4 (30.7)	9 (69.2)	
Site of tumor	Buccal mucosa	23 (65.7)	13 (56.5)	10 (43.4)	1.0
	Tongue	05 (14.2)	3 (60)	2 (40)	
	RMT	7 (20)	4 (57.1)	3 (42.8)	
Histological grade	WD	13 (37.1)	8 (61.6)	5 (38.4)	0.85
	MD	22 (62.9)	10 (45.4)	12 (54.5)	
	PD	0 (0)	0	0	
T-Stage	T1	10 (28.6)	3 (30)	7 (70)	0.21
	T2	9 (25.7)	6 (66.6)	3 (33.3)	
	T3	12 (34.3)	8 (66.6)	4 (33.3)	
	T4	4 (11.4)	1 (25)	3 (75)	
Nodal metastasis	N0	25 (71.4)	14 (56)	11 (44)	0.06
	N1	5 (14.3)	4 (80)	1 (20)	
	N2	3 (8.6)	0	3 (100)	
	N3	2 (5.7)	0	2 (100)	
LVI	Evident	11 (31.4)	6 (54.5)	5 (45.4)	0.80
	Not evident	24 (68.6)	12 (50)	12 (50)	
PNI	Evident	7 (20)	5 (71.4)	2 (28.5)	0.23
	Not evident	28 (80)	13 (46.4)	15 (53.5)	
Tumor budding	Evident	29 (82.9)	16 (55.1)	13 (44.8)	0.40
	Not evident	6 (17.1)	2 (33.3)	4 (66.6)	
WPOI	3	5 (14.3)	2 (40)	3 (60)	0.49
	4	17 (48.6)	8 (47)	9 (52.9)	
	5	13 (37.1)	8 (61.5)	5 (38.46)	
Depth of invasion	<5 mm	9 (25.7)	3 (33.3)	6 (66.6)	0.51
	5-10 mm	11 (31.4)	7 (63.6)	4 (36.3)	
	11-15 mm	10 (28.6)	6 (60)	4 (40)	
	>15 mm	5 (14.3)	2 (40)	3 (60)	
TILS	1+	7 (20)	3 (42.8)	4 (57.1)	0.87
	2+	17 (48.6)	9 (52.9)	8 (47)	
	3+	11 (31.4)	6 (54.5)	5 (45.4)	
Desmoplasia	1+	11 (31.4)	7 (63.6)	4 (36.3)	0.35
	2+	22 (62.9)	11 (50)	11 (50)	
	3+	2 (5.7)	0	2 (100)	
Tumor necrosis	Present	10 (28.6)	6 (60)	4 (40)	0.52
	Absent	25 (71.4)	12 (48)	13 (52)	
Pathological staging	Stage I	8 (22.9)	3 (37.5)	5 (62.5)	0.01*
	Stage II	7 (20)	4 (57.1)	3 (42.8)	
	Stage III	12 (34.3)	10 (83.3)	2 (16.6)	
	Stage IV	8 (22.9)	1 (12.5)	7 (87.5)	

The clinicopathologic parameters and CPS indicated a significant association with PNI and male gender; however, no other parameters showed a significant association (Table 3).

**Table 3:** Correlation of clinicopathological parameters and CPS of PD-L1 in OSCC The chi-square test/Fisher’s exact test was applied as appropriate. *P-value < 0.05 is considered significant. CPS, combined proportion score; WD, well differentiated; MD, moderately differentiated; PD, poorly differentiated; TILS, tumor infiltrating lymphocytes; T, tumor; LVI, lymphovascular invasion; PNI, perineural invasion; WPOI, worst pattern of invasion; OSCC, oral squamous cell carcinoma; PD-L1, programmed death-ligand 1

Features		Number, *n* (%)	CPS positive (*N* = 27), *n* (%)	CPS negative (*N* = 8), *n* (%)	*P*-value
Age intervals	≤40 years	9 (25.7)	6(66.6)	3(33.3)	0.39
	>40 years	26 (74.3)	21 (80.7)	5 (19.2)	
Gender	Male	22 (68.1)	21 (95.4)	1 (4.5)	<0.05*
	Female	13 (31.9)	6 (46.1)	7 (53.8)	
Site of tumor	Buccal mucosa	23 (65.7)	13 (56.5)	4 (17.3)	0.84
	Tongue	05 (14.2)	3 (60)	2 (40)	
	RMT	07 (20)	5 (71.4)	2 (28.5)	
Histological grade	WD	13 (37.1)	12 (92.3)	1 (7.6)	0.11
	MD	22 (62.9)	15 (68.1)	7 (31.8)	
	PD	0 (0)	0	0	
T-stage	T1	10(28.6)	7 (70)	3 (30)	0.76
	T2	9 (25.7)	7 (77.7)	2 (22.2)	
	T3	12 (34.3)	9 (75)	3 (25)	
	T4	4 (11.4)	4 (100)	0	
Nodal metastasis	N0	25 (71.4)	19 (76)	6 (24)	0.88
	N1	5 (14.3)	4 (80)	1 (20)	
	N2	3 (8.6)	2 (66.6)	1 (33.3)	
	N3	2 (5.7)	2 (100)	0	
LVI	Evident	11 (31.4)	9 (81.8)	2 (18.1)	0.65
	Not evident	24 (68.6)	18 (75)	6 (25)	
PNI	Evident	7 (20)	7 (100)	0	0.04*
	Not evident	28 (80)	20 (71.4)	8 (28.5)	
Tumor budding	Evident	29 (82.9)	23 (79.3)	6 (20.6)	0.29
	Not evident	6 (17.1)	4 (66.6)	2 (33.3)	
WPOI	3	5 (14.3)	3 (60)	2 (40)	0.48
	4	17 (48.6)	13 (76.4)	4 (23.5)	
	5	13 (37.1)	11 (84.6)	2 (15.3)	
Depth of invasion	<5mm	9 (25.7)	6 (66.6)	3 (33.3)	0.53
	5-10 mm	11 (31.4)	9 (81.8)	2 (18.1)	
	11-15 mm	10 (28.6)	7 (70)	3 (30)	
	>15 mm	5 (14.3)	5 (100)	0	
TILS	1+	7 (20)	7 (100)	0	0.12
	2+	17 (48.6)	12 (70.5)	5 (29.4)	
	3+	11 (31.4)	8 (72.7)	3 (27.2)	
Desmoplasia	1+	11 (31.4)	9 (81.8)	2 (18.1)	0.48
	2+	22 (62.9)	16 (72.7)	6 (27.2)	
	3+	2 (5.7)	2 (100)	0	
Tumor necrosis	Present	10 (28.6)	8(80)	2 (20)	0.79
	Absent	25 (71.4)	19 (76)	6 (24)	
Pathological Staging	Stage I	8 (22.9)	5 (62.5)	3 (37.5)	0.60
	Stage II	7 (20)	5 (71.4)	2 (28.5)	
	Stage III	12 (34.3)	10 (83.3)	2 (16.6)	
	Stage IV	8 (22.9)	7 (87.5)	1 (12.5)	

Survival analysis of cases

All the patients included in the study were last followed up in November 2023. The average survival duration was 15.28 ± 1.49 (mean ± standard error (SE)) months. Seven of the 35 patients had received chemotherapy, and four had received radiotherapy after the resection surgery. None of the patients had received combined chemoradiotherapy. Four patients died due to distant metastatic complications, and two patients died due to multiorgan failure. The PD-L1 CPS-positive and negative groups had comparable overall survival of 15.3 months and 16.5 months.

## Discussion

PD-L1 in OSCC has been evaluated in various studies. Before 2020, most studies used TPS to score PD-L1 at various cut-offs, including >1%, >5%, and >25% [13-15]. After 2020, many studies have used CPS for PD-L1 expression, as it has better therapeutic and clinical implications. In HNSCC, 1% CPS was considered positive, and CPS >20% was correlated better with treatment response [5,16-17].

In this study, we evaluated PD-L1 expression in OSCC using TPS and CPS, with positivity rates of 51.4% and 77.1%, respectively, comparable to previous studies [10,18]. The CPS score was higher in other studies, ranging from 85% to 89% [5,18]. The slightly lower positivity in our cases may be due to the small sample size. However, one study also reported a CPS positivity of 48%, with an overall range of 30%-80% [18-19]. We noted a significantly higher CPS in males, consistent with the findings from Gangadhar et al.'s Indian study [20]. This contrasts with the meta-analysis by Lenouvel et al., which reported higher rates of PD-L1-positive tumors in females [10].

A cut-off age of 40 years was set, and no significant association with PD-L1 expression emerged. This observation aligns with the study of Levounel et al., indicating no meaningful correlation between PD-L1 expression and age at various cutoff points (greater than 56, 60, and 65) [10]. However, in our cohort, the age group above >40 years was higher, and it is postulated that PD-L1 regulation is age-dependent, which suggests that the immunotherapy response is relatively reduced in elderly patients. We found the buccal mucosa to be the most frequent tumor site, with the highest proportion of PD-L1-positive cases (47%) originating from it. In contrast, previous research found that PD-L1-positive tumors predominantly occurred in the tongue, followed by buccal mucosa, showing higher TPS values [21-24].

We examined paired samples from pre-surgical biopsy and the subsequent surgical resection specimens without chemotherapy or radiotherapy. We found a low or negative PD-L1 expression in pre-surgical biopsy specimens, while resection specimens showed a trend of higher expression.

Ferris et al. and Keynote studies have challenged the use of a single tumor biopsy to measure PD-L1 expression for immunotherapy in head and neck SCC patients due to the ambiguous results caused by tumor heterogeneity [4,25].

Rasmussen et al. have observed that the TPS and CPS for PD-L1 expression remarkably vary within the tumor, limiting the utility of PD-L1 [26].

In this regard, it is noteworthy that in resection specimens with PD-L1 expression of ≥1% and ≥50% CPS, their corresponding small biopsies were regarded as negative expression, which supports the concept of intra-tumoral heterogeneity. Nevertheless, some studies have reported a good correlation between biopsy and surgical specimens [27]. In this study, we assessed the correlation between CPS and TPS and identified that in 18 cases, PD-L1 expression was seen only in tumor cells, and only TPS could be derived. On the other hand, in 27 cases, PD-L1 expression was seen in both tumor and immune cells, so that CPS could be derived in 27 cases. Gangadhar et al., a study conducted in India, reported similar results but utilized the 22C3 PD-L1 clone [20]. Our investigation reinforces that the SP263 clone can also effectively assess PD-L1 in OSCC.

Our findings indicate that 20 out of 27 CPS-positive cases showed higher immune cell positivity with PD-L1 intensity scores of +2/+3. Although this result was not statistically significant, it aligns with the theory that increased immune cells, by releasing inflammatory cytokines such as interferon-gamma (IFN-γ), may enhance the expression of PD-L1. This suggests a potentially better response to anti-PD-L1 treatment in tumors with elevated immune cells.

In the current research, TPS was found to have a significant correlation with staging, mainly with stage III tumors exhibiting elevated PD-L1 expression. In contrast, no notable relationship was established between T-stage or nodal metastasis in TPS and CPS scores, aligning with findings from Lenouvel et al., which reported a lack of significant association with T- or N-stage [21]. Nonetheless, a previous meta-analysis by Lenouvel et al. suggested that higher PD-L1 expression was observed in node-positive tumors when studies involved more than 50 patients [10].

In the current study, various histopathological parameters, such as PNI, LVI, tumor budding, WPOI, depth of invasion, desmoplasia, and tumor necrosis, were also evaluated in all the cases and correlated with PD-L1 expression (CPS and TPS). A notable correlation was found with PNI and CPS; however, other parameters showed no significant correlations, possibly due to the limited sample size. We could not identify any studies on PD-L1 expression in OSCC that included these parameters for comparison with our findings.

Our study revealed that the average survival rates for PD-L1 (CPS) positive and negative groups are similar; however, this conclusion cannot be overstated due to the restricted sample size. Prior meta-analyses have reported mixed survival outcomes, with one indicating no significant link between survival and PD-L1 expression. Conversely, Levounel et al. demonstrated that high levels of PD-L1 in OSCC were associated with poorer outcomes in both disease-specific survival (DSS) and disease-free survival (DFS) [10]. The varying findings regarding the prognostic importance of PD-L1 in different studies and meta-analyses require validation through larger sample sizes and standardized scoring methods before being incorporated into clinical practice.

Limitations

The current study's sample size was limited and derived from one tertiary care center, so the PD-L1 expression may not accurately reflect variations across different regions and ethnic groups.

## Conclusions

In conclusion, in the current study analyzing PD-L1 expression in OSCC using the SP263 assay, with both TPS and CPS, and comparing pre-surgical biopsy results to the subsequent surgical resection specimens, we observed a substantial difference in PD-L1 expression between the small biopsy and the paired larger resection specimens. This difference might be due to tumor heterogeneity. This underscores the clinical importance of small biopsies in evaluating PD-L1 immunostaining.

Our findings also reveal significant correlations between elevated CPS and male patients, as well as with evident PNI. These parameters could be used to stratify OSCC patients who may benefit substantially from emerging anti-PD-L1 therapies. However, it is important to note that studies with larger cohorts are needed for further evaluation and validation.
